# Supplementary material for: Coherent manipulation of a solid-state artificial atom with few photons
Source: Nat Commun. 2016 Jun 17;7:11986. doi: 10.1038/ncomms11986 (PMC4915012; doi:10.1038/ncomms11986)
Supplement: Supplementary Information — Supplementary Figures 1-2, Supplementary Notes 1-2 and Supplementary References [file ncomms11986-s1.pdf]

SUPPLEMENTARY FIGURE 1

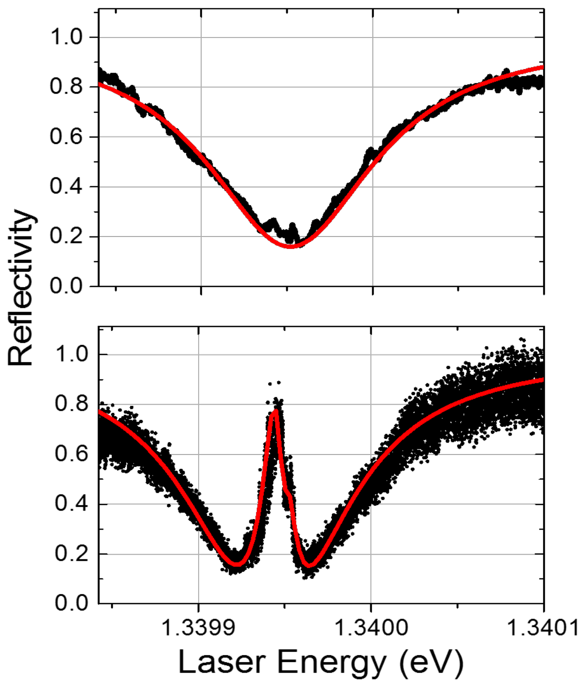

Supplementary Figure 1: **Reflectivity under continuous wave excitation.** Reflectivity spectra and relative fitting measured for a bias where the QD exciton transition is detuned from the cavity mode (top) or in resonance with the cavity mode (bottom).

SUPPLEMENTARY FIGURE 2

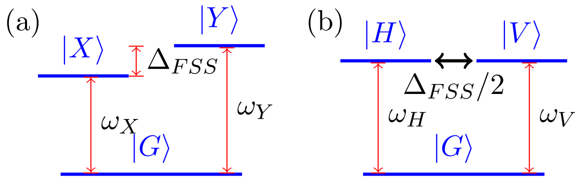

Supplementary Figure 2: **Schematic of the quantum dot states considered in the model:** (a) in the basis of the QD exciton eigenstates ($|X\rangle$,$|Y\rangle$); (b) in the ($|V\rangle$,$|H\rangle$) basis corresponding to the orientation of the cavity modes. For $\theta = \pi/4$ the coupling between $|V\rangle$ and $|H\rangle$ is $\Delta_{\mathrm{FSS}}/2$.

SUPPLEMENTARY NOTE 1: MEASUREMENTS

Sample

The microcavity sample was grown by molecular beam epitaxy. A λ-GaAs cavity is surrounded by a bottom and a top mirror made of 30 and 20 pairs of GaAs/$Al_{0.9}Ga_{0.1}As$. The bottom mirror is gradually n-doped while the top one is p-doped. After the in-situ optical lithography defining the cavities centered on the QDs, the sample is etched and standard p contacts are defined on a large frame ($\approx$ 300 $\times$ 300 μm^2) connected to the circular frame around the micropillar (see figure 1a and Ref. [1]). A standard n contact is defined on the back of the sample.

Experimental setups

The experimental setup is based on a confocal geometry where the same microscope objective (NA 0.75) serves simultaneously for quantum dot (QD) excitation and photoluminescence (PL) emission/collection. The excitation is provided by a tunable Ti-Sapph laser, providing 3 ps pulses at a 82 MHz repetition rate. The sample is kept at 4 Kelvin in a close-cycle cryostat with an exchange gas.

The laser pulse duration is adjusted by sending 3 ps laser pulses through a spectrometer and an etalon filter with 10 pm bandwidth. By adjusting the spectrometer slit size and the etalon angle, the pulse width can be continuously tuned between 10 ps and 100 ps.

Reflectivity measurement

To measure the reflectivity of the micropillar, a CW tunable laser focused on the pillar is reflected and sent to a detector (Exit 1 in figure 2a). The intensity of the reflected light is collected and measured as a function of the laser energy. When the exciton transition is not in resonance with the mode of the cavity, a reflectivity dip is observed corresponding to the bare cavity resonance (Supplementary Figure 1, top). The width of the cavity mode gives an accurate value of the cavity damping κ = 120 μeV corresponding to a Q-factor equal to 11000. From the value of the reflectivity at the cavity energy $R_{\min}$, we can extract the coupling of the pillar through the top mirror $\kappa_{\text{top}}/\kappa$: $R_{\min} = (1-2\kappa_{\text{top}}/\kappa)^2$. We extract two

possible values for the output coupling $\kappa_{\rm top}/\kappa = 0.7\pm0.05$ or $\kappa_{\rm top}/\kappa = 0.3\pm0.05$. Considering that the mirrors of the cavity are highly asymmetric (with 30/20 pairs) and that the same device acts as a bright single photon source [2], we deduce that $\kappa_{\rm top}/\kappa = 0.7 \pm 0.05$.

By changing the electrical applied bias, the exciton transition is tuned in resonance with the cavity mode. The exciton transition appears as the narrow peak in the middle of the cavity resonance (Supplementary Figure 1, bottom). The height of the peak depends on the coupling strength between the QD and the cavity (g) and on the coherence rate $\gamma = \gamma_{\rm sp}/2 + \gamma^*$, where $\gamma_{\rm sp}$ is the spontaneous emission rate in the other modes and γ^* the pure dephasing rate [3]. By fitting the experimental data, we extract a coupling strength equal to $g = 21\pm2$ μeV and a radiatively limited coherence rate equal to $\gamma = 0.30\pm0.10$ μeV. The device cooperativity $C = g^2/\kappa\gamma$ reaches the very high value of $C = 13$.

SUPPLEMENTARY NOTE 2: THEORY

The quantum dot (QD) is modeled as a three-level system with a ground state $|G\rangle$ and two excited states $|X\rangle$ and $|Y\rangle$ corresponding to two linearly polarized excitonic states (See Supplementary Figure 2). The respective energies of the excitons are denoted ω_X and $\omega_Y = \omega_X + \Delta_{\rm FSS}$ where $\Delta_{\rm FSS}$ is the fine structure splitting. The QD interacts with the two orthogonally polarized cavity modes H and V with respective lowering operators denoted a_H and a_V, of respective frequencies ω_H and ω_V. The exciton eigenstates are transformed into the cavity-polarization basis through a rotation by an angle θ: $|V\rangle = \cos(\theta)|X\rangle + \sin(\theta)|Y\rangle$ and $|H\rangle = -\sin(\theta)|X\rangle + \cos(\theta)|V\rangle$. In this basis, the Hamiltonian of the QD-cavity system reads

$$\hat{H}_{\rm s} = \hat{H}_{\rm QD} + \hat{H}_{\rm c} + \hat{H}_{\rm i}, \tag{1}$$

where $\hat{H}_{QD} = \hbar(\delta_V^{at}\sigma_V^\dagger\sigma_V + \delta_H^{at}\sigma_H^\dagger\sigma_H) + \Delta_{\rm FSS}\cos(\theta)\sin(\theta)(\sigma_H^\dagger\sigma_V + \sigma_V^\dagger\sigma_H)$ is the free Hamiltonian of the QD, and $\delta_V^{at} = \delta_X\cos^2(\theta) + \delta_Y\sin^2(\theta)$ and $\delta_H^{at} = \delta_X\sin^2(\theta) + \delta_Y\cos^2(\theta)$ where δ_X and δ_Y are detunings from the pump frequency, which we shall take as the reference. We have introduced $\sigma_V = |G\rangle\langle V|$ and $\sigma_H = |G\rangle\langle H|$. $\hat{H}_c = \hbar(\delta_V a_V^\dagger a_V + \delta_H a_H^\dagger a_H)$ is the free Hamiltonian of the cavity modes, and $\hat{H}_i = \hbar g(a_V\sigma_V^\dagger + a_H\sigma_H^\dagger + a_V^\dagger\sigma_V + a_H^\dagger\sigma_H)$ is the QD-cavity interacting term within the rotating-wave approximation.

Each exciton can relax towards the vacuum with a spontaneous emission rate γ. On the other hand, each cavity mode is coupled to a 1D continuum of modes and to a reservoir of lossy modes of same polarization, giving rise to a decay rate denoted κ and κ_{loss} respectively.

Excitation by a coherent field

When the cavity is pumped with a coherent V-polarized field through the 1D channel, it gives rise to an additional term in the Hamiltonian $\hat{H}_p = \hbar(\Omega^*(t)a_V + \Omega(t)a_V^\dagger)$ (see e.g. [4]). The time-dependent Rabi frequency reads $\Omega(t) = \sqrt{n\kappa}\xi(t)$, where n is the mean number of incident photons in the pulse, and the pulse $\xi(t)$ is a normalized Gaussian function checking

$$\xi(t) = \left(\frac{8\ln(2)}{\pi\tau^2}\right)^{1/4} \exp(-t^2/\tau^2 4\ln(2)). \tag{2}$$

Introducing ρ the density matrix of the coupled QD-cavity system, the Liouville equation ruling the dynamics writes:

$$\dot{\rho} = \mathcal{L}[\rho] = -\frac{i}{\hbar}\left[\hat{H}_s + \hat{H}_p, \rho\right] + D_{\gamma,\sigma_H}[\rho] + D_{\gamma,\sigma_V}[\rho] + D_{\kappa_{\text{tot}},a_H}[\rho] + D_{\kappa_{\text{tot}},a_V}[\rho], \tag{3}$$

where $D_{\alpha,X}[\rho] = \alpha\left(X\rho X^\dagger - \frac{1}{2}(X^\dagger X\rho + \rho X^\dagger X)\right)$ are Lindbladian superoperators describing the relaxation of the excitonic states with a rate γ and of the cavity modes with a total rate $\kappa_{\text{tot}} = \kappa + \kappa_{\text{loss}}$.

We have numerically solved this equation using the following parameters: $\hbar\delta_V = 0$, $\hbar\delta_H = -70\,\mu eV$, $\delta_X = -\Delta_{\text{FSS}}/2$, $\delta_Y = \Delta_{\text{FSS}}/2$, $\hbar\Delta_{\text{FSS}} = 15\,\mu eV$, $\hbar g = 21\,\mu eV$, $\hbar\kappa_{\text{tot}} = 120\,\mu eV$, $\kappa_{\text{loss}} = 0.3\,\kappa_{\text{tot}}$, $\hbar\gamma = 0.3\,\mu eV$, $\theta = \pi/4$. The time evolution of the excitonic population in the $|H\rangle$ and $|V\rangle$ modes are computed as $P_i(t) = \langle\sigma_i^\dagger\sigma_i\rangle$ with $i = H, V$. The flip probability is defined as the sum of the two exciton population $P_H(t)+P_V(t)$ taken at a time corresponding to its maximal value for a pulse with one photon on average. Finally the number of collected photons per pulse in the mode H is calculated as:

$$N_H = \kappa\int\langle a_H^\dagger a_H\rangle dt \tag{4}$$

Excitation by a single-photon Fock state

To account for an incoming single photon wave packet of the form $|1_\xi\rangle = \int dt\, \xi(t)\, a^\dagger\, |0\rangle$, we follow Ref. [5] and consider the following coupled master equations:

$$\begin{aligned}\dot{\rho}_{11} &= \mathcal{L}'[\rho_{11}] + \Omega(t)\left(\left[\rho_{01}, a_V^\dagger\right] - \left[\rho_{01}^\dagger, a_V\right]\right)\\ \dot{\rho}_{01} &= \mathcal{L}'[\rho_{01}] - \Omega(t)\,[\rho_{00}, a_V]\\ \dot{\rho}_{00} &= \mathcal{L}'[\rho_{00}],\end{aligned} \tag{5}$$

in which the generalized density matrices ρ_{mn} are defined as

$$\rho_{mn}(t) = \mathrm{Tr}_{\mathrm{ph}}\{U(t,t_0)\rho_s(t_0) \otimes |m_\xi\rangle\langle n_\xi| U^\dagger(t,t_0)\}, \tag{6}$$

$U(t,t_0)$ being the evolution operator corresponding to the total Hamiltonian from a time t_0 prior to the interaction. Hence ρ_{11} is the density matrix of the system when a single photon is incoming. The initial conditions are $\rho_{11}(0) = \rho_{00}(0) = \rho(0)$, $\rho_{01}(0) = 0$ and $\mathcal{L}'[\rho]$ is the Liouvillian defined by ρ:

$$\mathcal{L}'[\rho] = -\frac{i}{\hbar}\left[\hat{H}_s, \rho\right] + D_{\gamma,\sigma_H}[\rho] + D_{\gamma,\sigma_V}[\rho] + D_{\kappa_{\mathrm{tot}},a_H}[\rho] + D_{\kappa_{\mathrm{tot}},a_V}[\rho]. \tag{7}$$

Supplementary references

[1] Nowak, A. K. *et al.* Deterministic and electrically tunable bright single-photon source. *Nat. Commun.* **5**, 3240 (2014).

[2] Somaschi, N. *et al.* Near optimal single photon sources in the solid state. *Nature Photon.* **10**, 340–345 (2016). URL `http://dx.doi.org/10.1038/nphoton.2016.23` .

[3] Loo, V. *et al.* Optical nonlinearity for few-photon pulses on a quantum dot-pillar cavity device. *Phys. Rev. Lett.* **109**, 166806 (2012). URL `http://link.aps.org/doi/10.1103/PhysRevLett.109.166806`.

[4] Cohen-Tannoudji, C., Dupont-Roc, J. & Grynberg, G. *Atom-Photon interactions* (Wiley-VCH).

[5] Gheri, K., Ellinger, K., Pellizari, T. & Zoller, P. Photon-wavepackets as flying quantum bits. *Fortschritte der Physik* **46**, 401–416 (1998).
